# Real‐World Use of Rezafungin for the Treatment of Challenging *Aspergillus* Infections

**DOI:** 10.1111/myc.70230

**Published:** 2026-10-29

**Authors:** Neil Stone, Pablo Bachiller‐Luque, Simone Giuliano, Sumita Pai, Berta Pernas, Carolina Garcia‐Vidal, Netta Tyler, Sara Ferin, Carlo Tascini, Ana Fernández Cruz

**Affiliations:** ^1^ Hospital for Tropical Diseases/University College London Hospitals Infection Division London UK; ^2^ University Hospital Río Hortega Valladolid Spain; ^3^ Department of Medicine University of Udine and Azienda Sanitaria Universitaria Friuli Centrale Udine Italy; ^4^ Royal Papworth Hospital NHS Foundation Trust Cambridge UK; ^5^ Infectious Diseases Unit, A Coruña University Hospital/Clinic Virology Group, Biomedical Research Institute of A Coruña A Coruña Spain; ^6^ Infectious Diseases Department Hospital Clínic de Barcelona‐IDIBAPS, Universitat de Barcelona Barcelona Spain; ^7^ Infectious Diseases Unit Puerta de Hierro‐Majadahonda University Hospital, IDIPHISA Madrid Spain

**Keywords:** *Aspergillus*, azoles, echinocandin, mycoses, outpatient, pulmonary aspergillosis, rezafungin, triazoles

## Abstract

**Background:**

*Aspergillus* infections can pose significant treatment challenges, due in part to increasing triazole resistance. Clinical insight is needed to understand the potential for rezafungin, a next‐generation echinocandin with once‐weekly dosing, to address some limitations of current treatments for *Aspergillus* infections.

**Methods:**

This retrospective case series collected anonymized real‐world data from eight adults who received once‐weekly rezafungin for difficult‐to‐treat *Aspergillus* infections. The cases were provided by clinicians in Spain, the UK, and Italy with considerable experience in treating challenging fungal infections.

**Results:**

Most patients had pulmonary aspergillosis, nearly all had failed or were intolerant to triazole‐based regimens, and half had prior echinocandin or amphotericin B exposure. Rezafungin was used primarily to enable outpatient parenteral antimicrobial therapy (OPAT), avoiding the need for daily IV echinocandin in settings of azole resistance or intolerance. About half received rezafungin as monotherapy; others had combination regimens. Clinical and/or radiological improvement or disease stabilization was observed in most cases, including in patients with azole‐resistant infections. Notably, one patient achieved resolution of chronic otitis media, and others had negative cultures or sustained clinical benefit. Rezafungin was well tolerated, including in patients with renal impairment. One patient with chronic aspergilloma did not achieve adequate symptomatic control with rezafungin and required surgery.

**Conclusions:**

This report provides initial clinical insights on rezafungin as a potential option for difficult‐to‐treat *Aspergillus* infections. In this limited group of patients, rezafungin enabled OPAT, facilitated hospital discharge, and provided clinically meaningful improvements, including prolonged disease stabilization and resolution, which merit further investigation in clinical trials.

## 1 Introduction

Patients with *Aspergillus* infections can have significant disease burden including hypersensitivity (e.g., asthma and allergic bronchopulmonary aspergillosis), sinusitis, invasive aspergillosis, and chronic pulmonary infections [1, 2]. *Aspergillus* infections can be life threatening, particularly for immunocompromised patients. Globally, each year over 2,113,000 people develop invasive aspergillosis with mortality rates of 40%–80% [3]. Mould‐active triazoles are the cornerstone for treatment and prophylaxis of *Aspergillus* infections [4, 5, 6]. However, the effectiveness of triazoles is being challenged by the growing spread of azole‐resistant *Aspergillus* spp., particularly *A. fumigatus* , resulting in treatment failures and increased mortality rates [7, 8, 9]. In addition to *Aspergillus* spp. with intrinsic azole resistance, including newly identified ‘cryptic’ species, acquired resistance may develop with long‐term azole treatment due to selection of resistance mutations. While these are commonly observed in the *Aspergillus cyp51A* gene, other resistance mechanisms include loss‐of‐function mutations in regulatory genes leading to increased *cyp51A* expression, and over‐expression of drug efflux mechanisms [10, 11, 12]. Several resistance mechanisms may co‐exist within an isolate, further complicating antifungal management. Inhalation of azole‐resistant *Aspergillus* spores can also result in treatment failure in individuals without prior azole exposure [10].

Triazoles are associated with other challenges which can make them unsuitable for some patients. For example, triazoles have numerous drug–drug interactions (DDIs) which cause subtherapeutic or supratherapeutic exposure, or contraindications [13]. Therapeutic drug monitoring (TDM) is often required in patients receiving triazoles due to DDIs and highly variable pharmacokinetics (PK) [4, 14]. The tolerability profile can also be challenging for some patients. For example, long‐term use of triazoles is associated with hepatotoxicity, hormone‐related effects and adrenal suppression [15]. Phototoxicity and, in rare cases, skin malignancies are reported with voriconazole, while other serious side effects with some triazoles include peripheral neuropathy, pancreatitis, QT prolongation, and cardiac failure [15, 16].

Echinocandins, either as a single agent or in combination with an azole, are a recommended antifungal option for *Aspergillus* infections in settings that include azole resistance, as well as for prophylaxis and treatment of aspergillosis in patients undergoing haematopoietic stem cell transplantation (HSCT), or as targeted therapy for pulmonary disease [4, 5]. While echinocandins are fungicidal against pathogenic yeasts such as *Candida* species, fungistatic activity against *Aspergillus* species has limited their use as a primary treatment for severe *Aspergillus* infections [17]. However, due to a lower risk of side effects, echinocandins can be a useful treatment option for patients unable to tolerate triazoles or polyenes such as amphotericin B (AmB) which can cause nephrotoxicity and can be challenging to administer long term [18, 19, 20]. Furthermore, as echinocandins have a more predictable PK profile and fewer DDIs compared with triazoles and polyenes, the requirement for TDM is substantially lower [13].

Rezafungin is a next‐generation echinocandin with a broad spectrum of antifungal activity and distinct PK/pharmacodynamic profile. The low clearance and long half‐life of rezafungin enable once‐weekly intravenous (IV) dosing compared with daily IV dosing required for other echinocandins [21]. In addition to potent activity against most wild‐type and azole‐resistant *Candida* species and *Pneumocystis jirovecii*, rezafungin has broad activity against a range of *Aspergillus* species in vitro (including azole‐resistant strains) [22, 23]. Rezafungin was also shown to be effective for the treatment and prophylaxis of *A. fumigatus* infections in mouse models of invasive aspergillosis [24, 25].

Rezafungin is approved to treat adults with invasive candidiasis and candidaemia in countries including the US, Europe, Brazil, Saudia Arabia, and UAE. Approval is based on data from the Phase 3 ReSTORE (NCT03667690) and Phase 2 STRIVE (NCT02734862) trials which showed once‐weekly rezafungin was non‐inferior to daily caspofungin [26, 27]. Rezafungin is also being assessed in patients with chronic pulmonary aspergillosis and limited treatment options in a Phase 2 study (NCT06794554), and for prevention of invasive fungal diseases, including *Aspergillus* species, in adults undergoing allogeneic blood and marrow transplantation (ReSPECT: NCT04368559).

Real‐world data can support randomized clinical trials by providing insight into the potential utility of medicines across diverse clinical practice settings and highlight specific patient experiences. Following first approval of rezafungin in 2023, data are emerging on its real‐world use which provide insight on treatment effectiveness and challenges that can help inform clinical practice [28, 29, 30, 31]. In line with the licenced indication for rezafungin, real‐world cases published to date are focussed on patients with invasive candidiasis [28, 29, 30]. One recent case report described significant symptomatic improvement in a patient with long‐standing chronic pulmonary aspergillosis after 12 weeks of rezafungin monotherapy [31]. Here, we describe outcomes with rezafungin for eight patients with difficult‐to‐treat *Aspergillus* infections based on real‐world use.

## 2 Methods

Using a simple case report form, anonymous data were collected retrospectively on patients with challenging *Aspergillus* infections the authors had treated with off‐label rezafungin infusions (all patients received a loading dose of 400 mg on Day 1, followed by maintenance doses of 200 mg on Day 8 and weekly thereafter). The cases included *Aspergillus* infections that were associated with significant morbidity and had failed to respond to prior antifungal therapy, were associated with species harbouring azole‐resistance mutations, or if standard antifungal agents were not tolerated or contraindicated. All data were anonymized by the treating physician prior to analysis.

The following information was extracted from medical records: demographic characteristics (age [in years], sex, country), medical history relevant to the infection, *Aspergillus* infection details (diagnosis, species, minimal inhibitory concentration [MIC] data), prior antifungal agents and treatment duration, rationale for selecting rezafungin, duration of rezafungin therapy, concomitant antifungal agents, and clinical outcomes. The collated information was reviewed qualitatively for insights that may help inform clinical decision making for challenging *Aspergillus* infections.

## 3 Results

Data were available from eight adults (age range 27–78 years) with challenging *Aspergillus* infections who had received rezafungin. Demographic and infection characteristics, prior antifungal treatment, and outcomes with rezafungin for each patient are summarized in Table 1. A timeline of rezafungin administration and outcomes is shown in Figure 1.

**TABLE 1:** Summary of patients with *Aspergillus* infections treated with rezafungin.

	Patient 1	Patient 2	Patient 3	Patient 4	Patient 5	Patient 6	Patient 7	Patient 8
**Patient characteristics**								
Age (years)	72	46	42	44	78	43	27	57
Sex	F	F	F	F	M	M	F	F
Country	Spain	Italy	UK	Spain	Spain	UK	UK	Spain
Key medical history	Kidney transplantation complicated by rejection and TMA.	Recurrent otitis media (paediatric onset) and mastoiditis	Multiple sclerosis, AML	Severe asthma, bronchiectasis	COPD, lung cancer	Bronchiectasis, lobectomy/segmentectomy, recurrent lung infections, aspergilloma	Mediastinal B cell lymphoma	SLE, thymoma, Sjögren's syndrome, respiratory infection^b^
***Aspergillus* infection**								
Fungal species	*A. fumigatiaffinis*	*A. niger*	Nil cultured^e^	*A. fumigatus*	*A. fumigatus*	*A. fumigatus*	*A. fumigatus*	*A. fumigatus* ^c^
Diagnosis	Necrotic PA	Chronic/recurrent otitis media (left ear)	Invasive PA	Tracheo‐bronchial aspergillosis	PA	Chronic aspergilloma	Subacute invasive PA	PA
MIC (μg/mL)	Resistant to: AmB 8 VCZ 4 ISA 2 POS 0.25 Susceptible to: AND 0.03 CAS 0.12	ISA 2^a^ VCZ 2^a^ ITZ 2^a^ AND 0.5^a^ Susceptible to AmB 0.5	N/A	TR34/L98H mutation (azole resistant)	Susceptible to: AmB 0.38^g^ ITZ 0.38^g^ VCZ 0.19 POS 0.125 CAS 0.016 AND < 0.002 MICA 0.002 (*cyp51A* genotyping not performed)	Resistant to ITZ > 16 Susceptible to: AmB 0.5 AND < 0.015 ISA 0.25^f^ VCZ 0.25^f^ (*cyp51A* genotyping not performed^h^)	Susceptible to: LAmB, CAS Resistant to: ISA, VCZ, POS, ITZ (*cyp51A* genotyping not performed^h^)	Susceptible to: VCZ 0.125 ITZ 0.38 POS 0.064 ISA 0.25 AmB 0.5 (*cyp51A* genotyping not performed^h^)
Date of diagnosis	Jun 2022	Jan 2024	May 2024	Jun 2025	Aug 2023	2017	Apr 2025	Feb 2025
Prior antifungal treatment and duration (weeks)	ISA + AND (5) AND (11)	VCZ (8) LAmB (1.5) ISA + AND (1) ISA (8)	LAmB (4) ISA (4)	ISA (3)	VCZ (90)	VCZ (24) POS (28) POS + CAS (1)	POS (7) ISA (7)	None
**Rezafungin treatment**								
Indication	OPAT, azole resistance	OPAT, no response to prior antifungals, LAmB intolerance	LAmB/ISA intolerance, OPAT, mucositis	Azole resistance, OPAT	OPAT, radiological worsening	No response to prior antifungals	OPAT, azole resistance	OPAT
Monotherapy or combination	Monotherapy	+ISA	Monotherapy	+LAmB (inhaled)	+VCZ	Monotherapy, +LAmB (inhaled)	Monotherapy	+VCZ
Duration of rezafungin (weeks)	80	4	8	4	26	Monotherapy (2) +LAmB (4)	30 (ongoing)	8
Clinical response	Clinical improvement observed with AND was maintained with rezafungin	Resolution of otorrhea and pain	Stable disease	Clinical improvement (cough, dyspnoea, wheezing reduced). Negative mycobacterial culture of bronchial aspirate	Improved dyspnoea, asthenia, cough	Stable disease (with minor symptomatic improvement)	Stable disease	Improved dyspnoea. Sputum culture negative for *A. fumigatus* ^d^
Time to clinical response (weeks)	N/A	4	N/A	2	4	4	N/A	3–5 weeks
Radiological response	Reduced lesion size followed by stable disease	N/A	Stable disease	Bronchial abnormalities resolved; near complete resolution of tracheal lesions	Reduced lesion size and inflammation	Stable disease	Stable disease	Partial response: resolution of some pulmonary nodules
Time to radiological response (weeks)	10	N/A	N/A	4	4	N/A	8	8
Safety observations	Well tolerated	Well tolerated	Well tolerated	Well tolerated	Well tolerated	Well tolerated	Well tolerated	Well tolerated

Abbreviations: AmB, amphotericin B; AML, acute myeloid leukaemia; AND, anidulafungin; BAL, bronchoalveolar lavage; CAS, caspofungin; COPD, chronic obstructive pulmonary disease; F, female; GM, galactomannan; ISA, isavuconazole; ITZ, itraconazole; LAmB, liposomal amphotericin B; M, male; MIC, minimal inhibitory concentration; N/A, not applicable; NR, not reported; OPAT, outpatient antimicrobial therapy; PA, pulmonary aspergillosis; POS, posaconazole; SLE, systemic lupus erythematosus; TMA, thrombotic microangiopathy; VCZ, voriconazole.

^a^ Insufficient evidence for interpretive categories for *A. niger* with triazoles and echinocandins.

^b^ *Mycobacterium avium* , *Nocardia cyriacigeorgica, Scedosporium apiospermum*, and *Aspergillus fumigatus.*

^c^ Co‐infection with *Scedosporium apiospermum*.

^d^ Also negative for *Scedosporium apiospermum*.

^e^ Diagnosis based on positive serum galactomannan test.

^f^ MIC 0.5 μg/mL in a second sample.

^g^ MIC obtained using Etest strips.

^h^ Molecular sequencing was not a routine part of clinical diagnosis at the treating centre.

**FIGURE 1:**
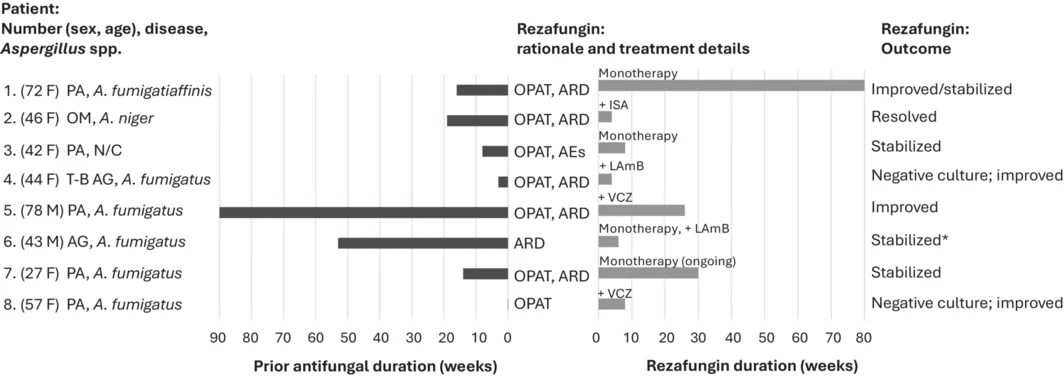
Overview of rezafungin administration and outcomes. *Minor symptomatic improvement and no radiological improvement; surgical intervention required. AE, adverse events; AG, aspergilloma; ARD, azole‐resistant disease; F, female; ISA, isavuconazole; LAmB, liposomal amphotericin B; M, male; N/C, nil cultured; OM, otitis media; OPAT, outpatient parenteral antimicrobial therapy; PA, pulmonary aspergillosis; T‐B AG, trachea‐bronchial aspergillosis; VCZ, voriconazole.

Four of the eight patient cases (Patients 1–4) are summarized in detail below. Details on Patients 5–8, which included some similar clinical themes to Patients 1–4, are provided in the Appendix and Figure S1.

### 3.1 Case Reports

#### 3.1.1 Patient 1

A 72‐year‐old female underwent kidney transplantation that was complicated by rejection and thrombotic microangiopathy, and received intensification of immunosuppressive therapy. Approximately 11 months later she was diagnosed with necrotic pulmonary aspergillosis caused by *A*. *fumigatiaffinis* (identified using β‐tubulin gene sequencing) that was resistant to azoles and AmB. Initial antifungal treatment (isavuconazole plus anidulafungin for 5 weeks) was switched to single‐agent anidulafungin based on in vitro susceptibility data which indicated susceptibility to echinocandins. Clinical improvement was observed within 48–72 h of initiating anidulafungin.

Following loss of the kidney graft, immunosuppressive therapy was discontinued and haemodialysis (with FX CorDiax 100 membrane) resumed. The patient became deconditioned during the prolonged hospitalization. To facilitate discharge, daily anidulafungin was switched to weekly rezafungin, administered as an outpatient. The patient received rezafungin for a total of 80 weeks. Clinical improvement observed with anidulafungin was maintained and further radiological improvement (via positron emission tomography–computed tomography [PET‐CT]) was observed after 10 weeks of rezafungin, followed by prolonged disease stabilization until her unrelated death (patient elected to stop haemodialysis) (Figure 2). Rezafungin was well tolerated throughout the treatment, including during haemodialysis.

**FIGURE 2:**
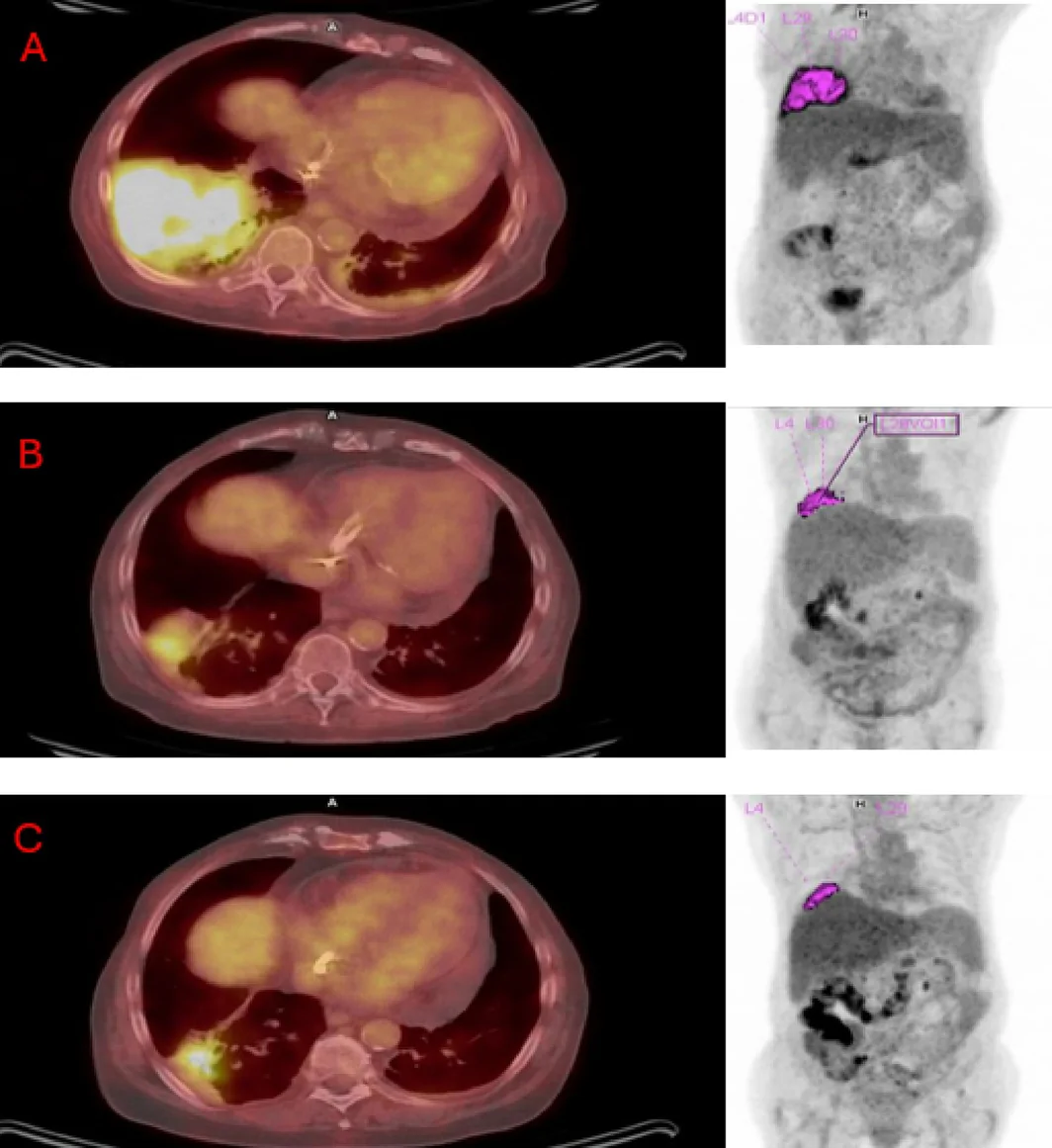
Radiological improvement of necrotic pulmonary aspergillosis. PET‐CT scans (Patient 1) of necrotic pulmonary aspergillosis showing increased tracer uptake 2 weeks after diagnosis (panel A), response after completing AND ± ISA (total 16 weeks) and 5 weeks of rezafungin (panel B), and further radiological improvement after 10 weeks of rezafungin (panel C). AND, anidulafungin; ISA, isavuconazole; PET‐CT, positron emission tomography–computed tomography.

#### 3.1.2 Patient 2

This 46‐year‐old female was diagnosed with mastoiditis (without CNS complications based on magnetic resonance imaging) and chronic/recurrent otitis media (left ear) since childhood. *A. niger* was isolated from a swab in January 2024. Otorrhea persisted despite an 8‐week course of voriconazole, and repeat swabs in June and August 2024 confirmed ongoing *A. niger* infection. In September 2024, the patient received liposomal AmB (LAmB) which was discontinued after 10 days due to acute nephrotoxicity (creatinine 2.93 mg/dL). In March 2025, the patient underwent left tympanoplasty with removal of granulation tissue to address ongoing symptoms; *A. niger* was confirmed in intraoperative cultures.

Immediately after surgery the patient received combination therapy with isavuconazole plus anidulafungin for 1 week, followed by isavuconazole monotherapy for 8 weeks. Given the refractory clinical course and persistent microbiological positivity, rezafungin was started in May 2025 based on its PK profile and suitability for combination therapy with isavuconazole (delayed initiation was due to challenges obtaining rezafungin and patient availability). Four once‐weekly rezafungin infusions were administered in the outpatient setting. The patient reported progressive clinical improvement and complete resolution of otorrhea and pain. Rezafungin plus isavuconazole combination therapy was well tolerated with no infusion‐related reactions, laboratory abnormalities or adverse events reported. The tympanoplasty site healed well and there were no signs of active infection following 4 weeks of isavuconazole plus rezafungin. Antifungal treatment was subsequently discontinued. At the 4‐month follow‐up the postoperative course was unremarkable, with no abnormal findings on clinical examination and an excellent functional outcome. No recurrence of infection was observed at the last assessment.

#### 3.1.3 Patient 3

A 42‐year‐old female with multiple sclerosis was diagnosed with invasive pulmonary aspergillosis in May 2024 while in remission from acute myeloid leukaemia. This diagnosis was based on positive serum galactomannan (optical density index 3.8 at diagnosis; confirmed in subsequent tests at later timepoints) and radiological findings (the culture was negative for infective species). The patient received LAmB for 4 weeks (with response) but, due to poor renal tolerance, was switched to isavuconazole. After 4 weeks, isavuconazole was stopped (the patient experienced deranged liver function tests and no response) and antifungal treatment was switched to rezafungin. Rezafungin was selected because the patient could no longer tolerate oral medications due to mucositis and to enable weekly IV OPAT at the haematology day unit. Rezafungin was well tolerated throughout 8 weeks of treatment and the disease remained stable. As there were no signals of clinical or radiological improvement and the patient was able to tolerate oral medications again, antifungal treatment was switched to posaconazole.

#### 3.1.4 Patient 4

This 44‐year‐old female with bronchiectasis and severe asthma (diagnosed age 20 years) that required repeat hospitalizations and systemic corticosteroids, was diagnosed with tracheo‐bronchial aspergillosis in June 2025. *A. fumigatus* was isolated from a sputum sample. Antifungal treatment with isavuconazole was started and the patient was discharged from hospital. Bronchoalveolar lavage shortly before discharge provided a borderline positive (0.76) galactomannan test, while PCR was positive for *A. fumigatus* with TR34/L98H mutation (identified using the AsperGenius multiplex qPCR assay), indicating azole resistance. Due to lack of clinical improvement after 3 weeks, antifungal treatment was switched to rezafungin plus nebulized LAmB. Rezafungin was selected due to the pan‐azole resistant infection, patient preference for a convenient dosing schedule, and lack of other well‐tolerated agents. Rezafungin plus LAmB was well tolerated. Clinical improvement, including reduction in cough, dyspnoea, and wheezing were seen from Week 2, and follow‐up bronchoscopy at Week 4 showed no bronchial abnormalities, near complete resolution of tracheal lesions, and mycobacterial culture of the bronchial aspirate was negative. Rezafungin plus LAmB was stopped and a week later tezepelumab was started as follow‐up treatment. The patient has sustained clinical improvement with no severe asthma exacerbations and tapering of oral prednisone.

### 3.2 Patient and Disease Characteristics

Most patients had pre‐existing lung disease, including bronchiectasis (*n* = 2), long‐standing respiratory infections (*n* = 2), and chronic obstructive pulmonary disease plus lung cancer (*n* = 1). Other significant medical history included haematological cancer (*n* = 2), kidney transplantation (*n* = 1), and recurrent otitis media (*n* = 1). Pulmonary aspergillosis (*n* = 5) was the most frequent *Aspergillus* infection diagnosis. Other diagnoses were chronic aspergilloma, trachea‐bronchial aspergillosis, and chronic otitis media with mastoiditis (*n* = 1 each).

Most infections were caused by *A. fumigatus* (*n* = 5). Other infective species were *A*. *fumigatiaffinis* and *A. niger* (*n* = 1, each). One patient with a negative culture was diagnosed with pulmonary aspergillosis based on a positive serum galactomannan test. Where available, MIC or mutation testing data indicated resistance to one or more triazole in over half the infections. Nearly all patients had received at least one line of triazole‐based antifungal therapy (as monotherapy or in combination with a daily echinocandin), including some with infection MIC data indicative of azole resistance, infection harbouring TR34/L98H azole‐resistant mutation, or cryptic species with intrinsic resistance (*A. fumigatiaffinis*). Two patients had received prior LAmB, and one had received single‐agent anidulafungin (Table 1).

The duration between diagnosis of the *Aspergillus* infection and starting rezafungin varied considerably. Rezafungin was administered approximately 1, 2, and 4 months after infection diagnosis for *n* = 2, *n* = 1, and *n* = 2 respectively, while this was 16 months, 2 years, and 7 years after diagnosis for *n* = 1, each.

### 3.3 Rezafungin Treatment Outcomes

In 7 of 8 patients, rezafungin was selected to enable OPAT due to the once‐weekly IV dosing schedule. Lack of response to triazole treatment (6 of 8 patients), including infection with *Aspergillus* species harbouring TR34/L98H or intrinsic resistance (*A. fumigatiaffinis*). Other reasons for selecting rezafungin included poor tolerance with other antifungal agents (triazoles and/or LAmB), and to continue antifungal therapy when swallowing difficulties due to mucositis prevented oral antifungal use.

Approximately half the patients received rezafungin as monotherapy, while combination therapy included triazole (voriconazole or isavuconazole) or LAmB. Rezafungin treatment resulted in disease improvements or disease stabilization for 7 of 8 patients. Of note, Patient 2 experienced resolution of long‐standing, recurrent otitis media after 4 weeks of rezafungin plus isavuconazole. Patient 4 (with *A. fumigatus* harbouring TR34/L98H) and Patient 8 achieved negative cultures and sustained clinical benefit after approximately 4 weeks of rezafungin combination therapy (rezafungin plus LAmB and rezafungin plus voriconazole, respectively). Patient 6, with chronic aspergilloma, did not respond adequately to rezafungin plus LAmB after 6 weeks (minor symptomatic improvement was observed with minimal radiological change) and rezafungin was discontinued (Table 1 and Appendix). Rezafungin was well tolerated by all patients, including Patients 2, 3 and 6 who were unable to tolerate triazoles and/or LAmB, and Patient 1 who received long‐term rezafungin treatment during haemodialysis.

## 4 Discussion

Challenges with standard‐of‐care triazoles include increasing emergence of resistant *Aspergillus* species, DDIs, and significant tolerance issues [4, 7, 15]. AmB‐based therapy also carries significant challenges in terms of toxicity, infusion reactions and a dosing schedule that makes outpatient therapy onerous [20]. Furthermore, species including *Aspergillus terreus* and *Aspergillus flavus* may exhibit intrinsic resistance or reduced susceptibility to AmB [32]. Echinocandins are an alternate treatment option, and while they are fungistatic rather than fungicidal against *Aspergillus* species, echinocandins are better tolerated with less propensity for DDIs and TDM than triazoles or polyenes [13, 17, 18]. However, providing daily IV echinocandin treatment outside of hospitalization is challenging. Indeed, several patients in this report were reluctant to prolong their hospital stay for daily IV antifungal treatment. Consequently, antifungal agents are needed that are convenient for patients as well as effective and well tolerated.

Using retrospective, real‐world data, we examined outcomes with rezafungin, a next‐generation echinocandin with unique once‐weekly dosing, across eight adults with challenging *Aspergillus* infections [21]. In this diverse group of patients, lung diseases were common. Other significant associated diseases included haematological cancer, kidney transplantation, and otitis media. Pulmonary aspergillosis caused by *A. fumigatus* was the most frequent infection. One patient had pulmonary aspergillosis caused by *A*. *fumigatiaffinis*, and one had chronic otitis media complicated by mastoiditis caused by *A. niger*. Prior to initiating rezafungin, nearly all patients had received at least one line of triazole‐based antifungal therapy without success, either as monotherapy or in combination with a daily echinocandin. Other prior lines of therapy included single‐agent anidulafungin and LAmB.

As well as a broad spectrum of antifungal activity, rezafungin has a distinct PK profile that enables once‐weekly IV dosing [22]. This extended dosing interval was the most frequent reason for initiating rezafungin in our patients, which enables OPAT by avoiding daily IV antifungal treatment. Recent real‐world reports indicate weekly OPAT with rezafungin decreases the treatment burden for patients with *Candida* infections versus daily IV echinocandin, with potential for cost savings [29, 33, 34]. While quality of life (QoL) was not assessed in our case series, studies show OPAT is associated with significant improvements in patient QoL compared with parenteral antimicrobial therapy in hospital [35, 36]. Other reasons for selecting rezafungin for our patients included triazole‐resistant disease (including lack of response to triazole treatment, infection with *Aspergillus* species harbouring TR34/L98H azole‐resistance mutation, or cryptic species with intrinsic resistance [*A. fumigatiaffinis]*), triazole and LAmB intolerance, and to continue antifungal therapy when mucositis prevented swallowing oral antifungals.

Rezafungin provided clinically meaningful improvements or disease stabilization for nearly all the patients with challenging *Aspergillus* infections. Resolution of long‐standing otitis media was noted in one patient after 4 weeks of rezafungin plus isavuconazole following no response to prior triazoles and AmB. Two additional patients had negative cultures and sustained clinical benefit after approximately 4 weeks of rezafungin combination therapy. This includes one individual with tracheo‐bronchial aspergillosis due to *A. fumigatus* harbouring TR34/L98H, tandem repeat in *cyp51A*, a mutation commonly associated with triazole resistance [12]. This observation aligns with a recent case report of a patient with chronic pulmonary aspergillosis caused by *A. fumigatus* with acquired triazole resistance (TR34/L98H mutation); this individual experienced symptomatic improvement as well as improved mood and weight gain after 12 weeks of rezafungin monotherapy [31]. Molecular sequencing was not performed for other patients in our case series with culture‐positive *A. fumigatus* infection as this was not a routine component of clinical diagnosis in the treating centres. However, wider use of *cyp51A* genotyping may improve insight into the cause for poor response to triazoles, including treatment‐emergent resistance. This includes Patient 7 in whom MIC data indicated pan‐triazole resistance and Patient 6 who had minimal response to posaconazole therapy for 28 weeks and known resistance to itraconazole.

In line with the fungistatic activity of echinocandins against *Aspergillus* species, most patients experienced some clinical and radiological improvements and/or disease stabilization rather than infection resolution. For complex *Aspergillus* infections, disease stabilization is often considered a positive outcome, particularly in settings of long‐term (salvage) antifungal therapy, as was observed with Patient 1. While current response definitions for clinical trials consider patients with stable disease to have failed antifungal therapy, there is a call for stable disease with continued antifungal treatment to be considered successful [37]. Rezafungin was not effective for all difficult‐to‐treat *Aspergillus* infections. One patient with chronic aspergilloma did not respond adequately to rezafungin plus LAmB after 6 weeks, and rezafungin was stopped. This patient had no response to 17 months of prior triazole‐based antifungal treatment, or single‐agent LAmB following rezafungin cessation, and required lobectomy to manage the aspergilloma (see Appendix). The timeframe between diagnosis of the challenging *Aspergillus* infection and onset of rezafungin treatment varied considerably across this group of patients (from 1 month to 7 years), suggesting potential opportunity for earlier initiation of effective antifungal treatment in some cases.

The safety profile of rezafungin in our patients is consistent with real‐world experiences of rezafungin for candidaemia and invasive candidiasis [28, 29, 30]. Rezafungin was well tolerated by all patients in this case series, including three individuals who did not tolerate triazoles and/or LAmB. While inhaled LAmB benefits some patients with azole‐refractory or ‐intolerant *Aspergillus* lung disease, this treatment is often poorly tolerated [38, 39]. In our patients, no concomitant medication adjustments were required to accommodate rezafungin, and no rezafungin‐related side effects were reported, including in a patient receiving haemodialysis and long‐term rezafungin monotherapy (for 80 weeks). Available data indicate rezafungin may be a potential antifungal option for patients with renal insufficiency. In contrast to azoles and some other echinocandins, available clinical data indicate rezafungin dose adjustments are unlikely be needed during continuous renal replacement therapy [40, 41]. Furthermore, outcomes with rezafungin were similar in patients with invasive candidiasis and moderate‐to‐severe renal impairment versus normal/mildly impaired renal function [40].

To date, while rezafungin is active against a range of *Aspergillus* species (including azole‐resistant strains) in vitro, clinical data are lacking [22]. Rezafungin use reported here reflects real‐world clinical decision making, where *Aspergillus* infection often lies on a spectrum between colonization and invasive disease, and treatment may be initiated when the pathogenic role is considered clinically plausible rather than strictly proven. These cases present new clinical observations about rezafungin for difficult‐to‐treat *Aspergillus* infections. While the retrospective nature, small number of patients, and potential for bias prevent our findings from being generalized, these observational findings may provide useful real‐world insights to support clinical trial data. This includes an ongoing Phase 2 trial assessing rezafungin in patients with chronic pulmonary aspergillosis and limited treatment options (NCT06794554). ReSPECT, a recently completed Phase 3 study, is investigating rezafungin in adults undergoing allogeneic blood and marrow transplantation (NCT04368559). Data from this study may provide insight into the effectiveness of rezafungin for preventing breakthrough infections with *Aspergillus* (and other fungal species) compared with the antimicrobial standard of care.

In summary, to our knowledge, our findings provide clinical evidence for the potential use of rezafungin for difficult‐to‐treat *Aspergillus* infections and merit further investigation in clinical trials. These cases suggest rezafungin may have utility for patients with *Aspergillus* infections experiencing triazole failure or intolerance. Importantly, the extended dosing schedule enabled hospital discharge and OPAT, allowing patients to be at home between outpatient visits. OPAT with rezafungin for challenging *Aspergillus* infections has potential for cost savings. A case report of rezafungin monotherapy for 12 weeks in a patient with triazole‐resistant chronic pulmonary aspergillosis found a small increase in OPAT cost compared with daily caspofungin (due to higher drug acquisition cost) was offset by decreased travel to the clinic, optimized nursing time, and increased clinic capacity with rezafungin [31]. However, health economic assessment, alongside low propensity for side effects and DDIs, and lack of requirement for TDM requires validation in larger studies of rezafungin in this setting.

## Author Contributions

**Neil Stone:** data curation, writing – review and editing, supervision; project administration; resources. **Pablo Bachiller‐Luque:** writing – review and editing; resources; data curation. **Simone Giuliano:** writing – review and editing; resources; data curation. **Sumita Pai:** writing – review and editing; resources; data curation. **Berta Pernas:** writing – review and editing; resources; data curation. **Carolina Garcia‐Vidal:** writing – review and editing; resources; data curation. **Netta Tyler:** writing – review and editing; resources; data curation. **Sara Ferin:** writing – review and editing; resources; data curation. **Carlo Tascini:** writing – review and editing; resources; data curation. **Ana Fernández Cruz:** writing – review and editing; resources; data curation.

## Funding

Medical writing support was funded by Mundipharma Research Ltd (UK).

## Ethics Statement

Ethical approval was not required for this non‐interventional, retrospective analysis of anonymized observational data. Patient anonymity was maintained throughout. The authors confirm that the ethical policies of the journal, as noted on the journal's author guidelines, have been adhered to. Patients provided permission for their anonymized clinical information being published in this manuscript (one individual died prior to the development of this paper and contacting their next of kin was not feasible).

## Conflicts of Interest

Neil Stone reports speaker fees from Mundipharma/Napp Pharmaceuticals. Pablo Bachiller‐Luque reports support for meeting attendance from Gilead. Simone Giuliano reports consulting fees from Basilea, Mundipharma, and Viatris; and honoraria from Advanz Pharma, Angelini, Gilead, Hikma, Infectopharm, Merck, Menarini, Mundipharma, Novartis, Pfizer, and Shionogi. Sumita Pai has no potential conflicts of interest to disclose. Berta Pernas reports support for meeting attendance from Mundipharma. Carolina Garcia‐Vidal reports honoraria from Advanz Pharma, bioMérieux, Gilead Sciences, MSD, Mundipharma, Pfizer, Shionogi, and Viatris; and grant support from Gilead Sciences and Mundipharma. Netta Tyler reports consulting fees from Napp Pharmaceuticals; honoraria from Napp Pharmaceuticals and Pfizer; and support for meeting attendance from the British Society for Antimicrobial Chemotherapy, Gilead, Napp Pharmaceuticals, and Tillomed Laboratories. Sara Ferin has no potential conflicts of interest to disclose. Carlo Tascini reports honoraria from Mundipharma. Ana Fernández Cruz reports honoraria and support for meeting attendance from Gilead, Pfizer, and Mundipharma.

## Supporting information


**Figure S1:** CT scans (Patient 5) showing a complex lung lesion with interstitial infiltrates and small nodules (< 1 cm) after a 3‐month course of chemotherapy and during treatment with voriconazole (A–C); corresponding CT images obtained following 1 month of rezafungin plus voriconazole showed reductions in both size of lesions and inflammatory changes (D–F).

**Data S1:** Supporting information.

## Data Availability

The data that support the findings of this study are available on request from the corresponding author. The data are not publicly available due to privacy or ethical restrictions.
