# Supplementary material for: Real‐World Use of Rezafungin for the Treatment of Challenging Aspergillus Infections
Source: Mycoses. 2026 Oct 29;69(11):e70230. doi: 10.1111/myc.70230 (PMC13717284; doi:10.1111/myc.70230)
Supplement: Supplementary file 1 [file MYC-69-e70230-s002.pdf]

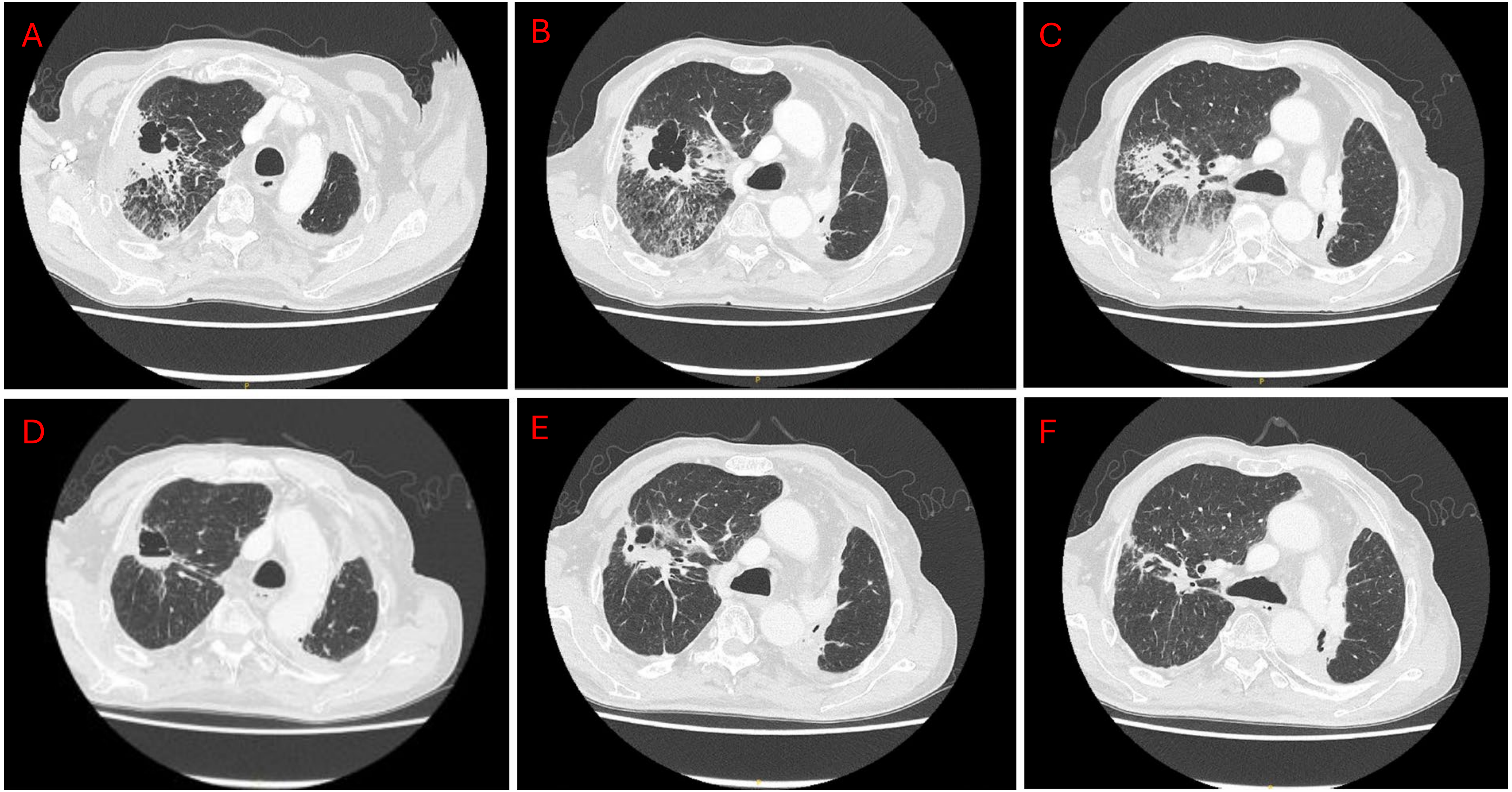

Figure S1. CT scans (Patient 5) showing a complex lung lesion with interstitial infiltrates and small nodules (< 1cm) after a 3-month course of chemotherapy and during treatment with voriconazole (**A–C**); corresponding CT images obtained following 1 month of rezafungin plus voriconazole showed reductions in both size of lesions and inflammatory changes (**D–F**)
