# Supplementary material for: Real‐World Use of Rezafungin for the Treatment of Challenging Aspergillus Infections
Source: Mycoses. 2026 Oct 29;69(11):e70230. doi: 10.1111/myc.70230 (PMC13717284; doi:10.1111/myc.70230)
Supplement: Supplementary file 2 [file MYC-69-e70230-s001.docx]

# Real-world use of rezafungin for the treatment of challenging *Aspergillus* infections

## Supplementary appendix. Case summaries: Patients 5–8

## Patient 5

A 78-year old male had a history including chronic obstructive pulmonary disease and squamous cell lung cancer (treated initially at diagnosis in 2011 with neoadjuvant chemotherapy, lobectomy and lymphadenectomy, which relapsed in 2012 and was treated with chemotherapy and radiotherapy]). A complex lung lesion (cystic areas with irregular borders, thickened walls, and ground-glass opacity) with increased tracer uptake was noted via positron emission tomography–computed tomography (PET-CT) in 2022 (biopsies were inconclusive for malignancy). In August 2023 the patient reported shortness of breath, weakness, and dry cough. Following routine bronchoscopy, *Aspergillus fumigatus* (susceptible to triazoles, echinocandins, and amphotericin B [AmB]; *cyp51A* genotyping was not performed) was isolated in bronchial aspirate and bronchoalveolar lavage (BAL); galactomannan was negative in serum and BAL, and serum 1,3-β-D-glucan was also negative. Voriconazole was initiated and provided symptomatic improvement. In January 2024, a follow-up PET-CT scan showed the complex cystic lung lesion was stable and bronchoscopy remained inconclusive for malignancy or infection; voriconazole was continued. However, in January 2025, bronchoscopy confirmed an undifferentiated lung cancer and bronchoalveolar lavage (BAL) galactomannan test was positive (1.5). Chemotherapy was started in February 2025. A CT scan 3 months later showed the lung mass had reduced in size but with worsening inflammation, new interstitial infiltrates, and nodules <1 cm, suggesting infection. Chemotherapy was stopped. As the patient lived too far from the hospital to permit daily outpatient treatment with an IV antifungal agent, rezafungin was selected in combination with voriconazole to treat refractory aspergillosis. Weekly rezafungin infusions were administered at the primary care centre (the first dose was provided in hospital). After 1 month of rezafungin plus voriconazole, the complex cystic lung lesion had decreased in size considerably and in inflammatory changes were reduced. Figure S1 shows CT scans obtained during treatment with single-agent voriconazole (panels A–C) and after 1 month of rezafungin plus voriconazole (panels D–F). Progressive symptomatic improvement (reduced cough, dyspnoea, and asthenia) was seen from Week 4, and rezafungin plus voriconazole was well tolerated. Antifungal treatment was stopped after 6 months following a CT scan which showed further improvement in cystic areas but an increasing solid component, attributed to the malignancy.

## Patient 6

This 43-year old male had undergone lobectomy/segmentectomy in 2018 for cavitating infection with *M. kansasii* and also had bronchiectasis and recurrent lung infections, and aspergilloma since 2017. Due to symptom burden, worsening lung function, and *Aspergillus* precipitins, the aspergilloma was initially treated with high-dose voriconazole which was not tolerated. Despite lowering the dose the patient experienced photosensitivity, nausea, malaise, and deranged liver function tests. Following prolonged treatment with voriconazole (24 weeks) there was no evidence of radiological improvement and antifungal treatment was switched to posaconazole.

Posaconazole was continued for 28 weeks, with the addition of caspofungin for 1 week to treat a possible fungal abscess noted via CT, and concern regarding potential development of subacute invasive disease. Due to minimal clinical and radiological improvement (posaconazole resistance was suspected [minimal inhibitory concentration data for posaconazole were unavailable]), antifungal treatment was switched to rezafungin. Rezafungin was selected to enable echinocandin-based outpatient antimicrobial therapy (OPAT), with addition of nebulised liposomal amphotericin B (LAmB) after 2 weeks of single-agent treatment. At Week 4 of the treatment course, some minor symptomatic improvement, including increased walking and marginal improvement in lung function, was noted while there was minimal radiological change. Rezafungin was stopped after 6 weeks and the patient continued to receive single-agent LAmB for 19 weeks. Due to ongoing symptoms, the patient underwent lobectomy to manage the aspergilloma from which *A. fumigatus* was isolated (resistant to itraconazole, susceptible to AmB, anidulafungin [and caspofungin, by extrapolation], voriconazole, and isavuconazole; *cyp51A* genotyping was not performed).

## Patient 7

This 27-year old female with mediastinal B cell lymphoma was diagnosed with subacute invasive aspergillosis in April 2025 caused by *A. fumigatus*. Diagnosis was based on positive BAL culture and galactomannan, and lung cavities noted on a CT scan. Posaconazole was given for 7 weeks and then changed to isavuconazole due to clinical and radiological deterioration. As susceptibility testing showed a triazole-resistant infection (*cyp51A* genotyping was not performed), antifungal treatment was switched to rezafungin. Rezafungin was selected due to more convenient dosing than LAmB, and to allow OPAT at the haematology day unit via weekly infusions. A CT scan after 8 weeks of rezafungin monotherapy showed disease stabilization, and clinical symptoms remained stable. Rezafungin treatment is ongoing with continued stable disease and has been well tolerated during the 7 months treatment duration, to date.

## Patient 8

This 57-year old female had a history including Sjögren's syndrome, thymoma (Stage IIa), systemic lupus erythematosus (recently treated with corticosteroids), and respiratory infection with *Mycobacterium avium* (ongoing treatment [rifampicin, ethamburol, and azithromycin] since April 2024). In December 2024, *Nocardia cyriacigeorgica, Aspergillus fumigatus,* and *Scedosporium apiospermum* were noted in bronchoscopy samples, and the patient’s symptoms improved with ceftriaxone to treat *Nocardia cyriacigeorgica*. However, respiratory function worsened in February 2025, and a follow-up bronchoscopy sample confirmed ongoing infection with *Aspergillus fumigatus* (susceptible to voriconazole, itraconazole, posaconazole, isavuconazole, and AmB [*cyp51A* genotyping was not performed]).and *Scedosporium apiospermum*. Worsening dyspnoea progressed to respiratory failure and there was little improvement with bronchodilator and intensified corticosteroid treatment. The patient had a strong preference to avoid prolonged hospitalization and daily hospital visits for antifungal treatment were not feasible. Consequently, antifungal treatment with rezafungin was started in March 2025, with weekly infusions provided as an outpatient. Rezafungin was given in combination with voriconazole to provide synergy in treatment, particularly for *Scedosporium apiospermum*. To avoid drug–drug interactions with voriconazole, rifampin was switched to clofazimine. Symptomatic improvement, including reduced dyspnoea, was seen after 3–5 weeks of starting rezafungin plus voriconazole and the patient remained afebrile. Antifungal treatment was well tolerated, and improvement in chronic mucositis was noted. A PET-CT scan indicated partial radiological response, with disappearance of some pulmonary nodules previously observed (there was no significant morphological change in the remaining nodules). Rezafungin plus voriconazole was stopped after 8 weeks. *Pseudomonas aeruginosa* was isolated in follow-up sputum samples and treated with ciprofloxacin. Two subsequent sputum samples were negative for *Aspergillus fumigatus* and *Scedosporium apiospermum*; treatment for *Mycobacterium avium* infection is ongoing.
